# Markers of oxidative stress during post-COVID-19 fatigue: a hypothesis-generating, exploratory pilot study on hospital employees

**DOI:** 10.3389/fmed.2023.1305009

**Published:** 2023-12-04

**Authors:** Hanna Hofmann, Alexandra Önder, Juliane Becker, Michael Gröger, Markus M. Müller, Fabian Zink, Barbara Stein, Peter Radermacher, Christiane Waller

**Affiliations:** ^1^Department of Psychosomatic Medicine and Psychotherapy, General Hospital Nuremberg, Paracelsus Medical University, Nuremberg, Germany; ^2^Anesthesiological Pathophysiology and Process Engineering, University Hospital, Ulm, Germany

**Keywords:** reactive oxygen species (ROS), oxidative stress, oxidative DNA damage, mitochondrial dysfunction, post-COVID-19 fatigue

## Abstract

**Introduction:**

Post-COVID-19 fatigue is common after recovery from COVID-19. Excess formation of reactive oxygen species (ROS) leading to oxidative stress-related mitochondrial dysfunction is referred to as a cause of these chronic fatigue-like symptoms. The present observational pilot study aimed to investigate a possible relationship between the course of ROS formation, subsequent oxidative stress, and post-COVID-19 fatigue.

**Method:**

A total of 21 post-COVID-19 employees of the General Hospital Nuremberg suffering from fatigue-like symptoms were studied during their first consultation (T1: on average 3 months after recovery from COVID-19), which comprised an educational talk on post-COVID-19 symptomatology and individualized outpatient strategies to resume normal activity, and 8 weeks thereafter (T2). Fatigue severity was quantified using the Chalder Fatigue Scale together with a health survey (Patient Health Questionnaire) and self-report on wellbeing (12-Item Short-Form Health Survey). We measured whole blood superoxide anion (${\text{O}}_{2}^{•-}$) production rate (electron spin resonance, as a surrogate for ROS production) and oxidative stress-induced DNA strand breaks (single cell gel electrophoresis: “tail moment” in the “comet assay”).

**Results:**

Data are presented as mean ± SD or median (interquartile range) depending on the data distribution. Differences between T1 and T2 were tested using a paired Wilcoxon rank sign or *t*-test. Fatigue intensity decreased from 24 ± 5 at T1 to 18 ± 8 at T2 (*p* < 0.05), which coincided with reduced ${\text{O}}_{2}^{•-}$ formation (from 239 ± 55 to 195 ± 59 nmol/s; *p* < 0.05) and attenuated DNA damage [tail moment from 0.67 (0.36–1.28) to 0.32 (0.23–0.71); *p* = 0.05].

**Discussion:**

Our pilot study shows that post-COVID-19 fatigue coincides with (i) enhanced ${\text{O}}_{2}^{•-}$ formation and oxidative stress, which are (ii) reduced with attenuation of fatigue symptoms.

## 1 Introduction

Fatigue after acute viral infection is a well-known consequence of, e.g., an Ebstein-Barr virus (EBV) infection (1). Similarly, after the acute infection with SARS-CoV-2 has resumed, a significant number of patients are continuously suffering from various physical and psychological symptoms, eventually lasting for several months (2), among which post-infectious fatigue is a common finding (3). Fatigue is characterized by severe physical and mental exhaustion disproportionate to the previous activity (2), which results in markedly impaired cardiorespiratory fitness (4). In post-COVID-19 patients, female sex and a pre-existing diagnosis of depression and/or anxiety are frequently present (5), while the degree of fatigue is often unrelated to the initial disease severity (5, 6). Despite the high impact on individual mental and physical health and quality of life, the pathophysiology of this fatigue is still not known (7).

Post-COVID-19 fatigue symptomatology resembles that of myalgic encephalomyelitis/chronic fatigue syndrome (ME/CFS) (8), and substantial overlap has been reported between post-COVID-19 and ME/CFS symptoms (9). Persistent neuroinflammation (10) and brain antioxidant capacity (11), redox imbalance (oxidative stress) (12), and consecutive mitochondrial dysfunction resulting from impaired mitochondrial respiratory activity and/or a reduced number of intact mitochondria (13) have been referred to as a possible link between post-COVID-19 fatigue and ME/CFS. Most recently, a significant relationship was shown between a neuropsychiatric symptoms score and a score based on the relationship between serum markers of oxidative and nitrosative stress and antioxidant capacity (14). Finally, oxidative stress is defined as the mismatch between the production and/or accumulation of reactive oxygen species (ROS) and the radical scavenger (antioxidant) capacity (15). This can result in damage to the DNA and/or mitochondria, the latter being mainly responsible for cellular energy metabolism. ROS formation is a natural process (16), e.g., for antimicrobial host defense (17), and mitochondrial respiration is the major source of ROS generation (18).

Activated immune cells (monocytes, neutrophils) also directly release ROS through NADPH oxidase activity (19). However, this excess ROS formation has also been referred to as a major pathophysiological mechanism of COVID-19: by increasing extracellular trap formation, it suppresses the T-cell response, i.e., the adaptive immune system response necessary to eliminate virus-infected cells (20).

Given the fundamental role of oxidative stress during the acute phase of a SARS-CoV-2 infection, we aimed to assess a possible relationship between oxidative stress and sequelae in patients who had recovered from the disease. For this purpose, in the present hypothesis-generating, exploratory pilot study, we investigated markers of oxidative stress and post-COVID-19 fatigue symptoms in hospital employees. We collected psychosocial data and analyzed ROS concentration and oxidative DNA damage in blood cells at two different time points prior to and after psychosomatic counseling.

## 2 Methods

### 2.1 Subjects and ethics

The present dataset is based on data collected from 21 hospital employees of the post-COVID-19 outpatient clinic at the Department of Psychosomatic Medicine and Psychotherapy, General Hospital Nuremberg, Paracelsus Medical University. The outpatient clinic was set up in March 2021 to support healthcare workers in the metropolitan region of Nuremberg in dealing with the consequences of a SARS-CoV-2 infection and to initiate treatment if necessary.

Prior to inclusion, all subjects gave their written informed consent for participation. The study was conducted in accordance with the Declaration of Helsinki; the study protocol had been approved by the Ethics Committee of the Paracelsus Medical University (No. FMS_W_010.22-XI-3) and the Bavarian State Chamber for Physicians (Bayrische Landesärztekammer No. 22035) and registered in the German Registrary for Clinical Studies (ID: DRKS00028108).

### 2.2 Study design

The present observational, hypothesis-generating clinical pilot study was carried out on patients of the interdisciplinary post-COVID-19 consultation hour established at General Hospital Nuremberg for hospital employees of all professional groups. Inclusion criteria were age between 18 and 70 years, COVID-19 infection, fatigue symptomatology, and post-COVID-19 syndrome according to the “Long/Post-COVID” guideline of the “*Arbeitsgemeinschaft der Wissenschaftlichen Medizinischen Fachgesellschaften*” (AWMF) (21). Exclusion criteria were insufficient knowledge of the German language to answer the questionnaires, an untreated somatic disease susceptible to provoking fatigue-like symptoms (e.g., malnutrition, electrolyte disturbances, and endocrine and neurological disorders), and/or the presence of a psychiatric disorder (such as addictive disorder, dementia, psychotic disorder, or suicidality). In particular, except for three individuals, none of the patients included had undergone psychotherapy within 12 months preceding the SARS-CoV-2 infection. A total of 16 women and 5 men with a median age of 52 (range: 32–64) years were recruited. The acute SARS-CoV-2 infection occurred between March 2020 and December 2021; the time interval between the SARS-CoV-2 infection and the first visit (T1) to the interdisciplinary post-COVID-19 consultation was at least 3 months. In 20 out of the 21 patients, SARS-CoV-2 treatment was confined to outpatient clinical care; the only patient requiring hospitalization did not need any ICU treatment. Hence, the patients studied had only shown mild to moderate severity of the acute SARS-CoV-2 infection; long-term pulmonary and/or cardiovascular sequelae were not present either.

Employees with fatigue symptoms presented at the Department of Psychosomatic Medicine and Psychotherapy between 10 a.m. and 12 p.m. for about half an hour and were always treated by the same physician (C.W.). Before the consultation started, the participants were asked to fill out the questionnaires. This was followed by a medical history interview. After a rest period of 5 min, blood was taken and immediately processed at a mobile lab desk for analysis of reactive oxygen species (ROS) formation and oxidative DNA damage. The intervention consisted of an educational talk during which the clinician explained the typical symptoms of the post-COVID-19 syndrome and the relationship between both physical and psychosocial stress and symptom amplification in the recovery phase. Depending on the degree of stress, an individualized outpatient procedure was determined to allow for the resumption of everyday and work activities, and a second psychosomatic consultation was arranged at an interval of 8 weeks to assess the progress (T2). At T2, the completion of the questionnaires and blood sampling were carried out in the same way as at T1. For the first counseling, the total data of 21 employees were analyzed, while for the second examination, only 15 employees took the service: one person could not have a blood draw, and five others did not need a second conversation; therefore, their questionnaire data are missing for T2.

### 2.3 Psychometric analysis

In addition to the collection of the sociodemographic data “age,” “gender,” and “time and course of SARS-CoV-2 infection,” the following psychometric analyses were performed:

#### 2.3.1 Mental health

Mental health was surveyed using the German Version of the Patient Health Questionnaire (PHQ-D) (22) which is a self-assessment tool consisting of several modules. We used the PHQ-D modules “somatization (PHQ-15),” “depression (PHQ-9),” and “stress (PHQ-Stress).” The PHQ-15 includes 15 physical complaints such as abdominal pain, headache, dizziness, shortness of breath, or palpitations. Respondents are asked to indicate to what extent they feel affected by the symptoms mentioned during the last 2 and 4 weeks for lack of energy and sleep disorder, respectively. The PHQ-9 module on depression comprises nine items. Participants are asked how often they felt affected by complaints like loss of interest, hopelessness, reduced appetite, or concentration difficulties during the last 2 weeks. The “PHQ-stress” measures psychosocial stress factors comprising, 10 items. For example, it asks how much a person felt affected by worries about their health, difficulties with their partner, stress at work, or financial worries during the last 4 weeks. The response formats are as follows: For PHQ-15 and PHQ-Stress, *0* = not bothered at all, *1* = bothered a little, and *2* = bothered a lot, and for PHQ-9, *0* = not at all, *1* = several days, *2* = more than half the days, and *3* = nearly every day. The evaluation of the individual modules is done by forming the sum value. For PHQ-15, this can range from 0 to 30; for PHQ-9, from 0 to 27; and for PHQ-Stress, from 0 to 20. Higher total scale values indicate a more severe mental disorder. Scale sum scores can be categorized and interpreted as follows: minimal (0–4), mild (5–9), moderate (10–14), and severe (≥15); for PHQ-9, moderate (10–14), moderately severe (15–19), and severe (≥20) symptom expression.

#### 2.3.2 Self-report of health and wellbeing

The German version of the “Short-Form-12 Health Survey” (SF-12) (23) was used to measure health-related quality of life. The SF-12 is a short version of the Short-Form-36 Health Survey (SF-36) (24) and consists of 12 items. The eight dimensions of the SF-36 are represented in the SF-12 by four individual items (general health perception, pain, vitality, and social functioning) and four item pairs (physical functioning, physical role functioning, emotional role functioning, and psychological wellbeing). Respondents are asked to use multilevel response scales to describe, e.g., their health in general (*1* = excellent to *5* = poor), to assess whether and if so, to what extent, they had been limited by their current health in moderately difficult activities (e.g., moving a table, vacuuming, bowling, playing golf; *1* = yes, severely limited to *3* = no, not limited at all), or, e.g., how often they had felt “*full of energy*” in the past 4 weeks (*1* = always to *6* = never). The subscales of general perception of health, physical functioning, physical role functioning, and pain represent the physical dimension of health. Vitality, psychological wellbeing, emotional role function, and social functioning represent the psychological dimension. A sum scale can be calculated for both physical (Physical Composite Score) and mental (Mental Composite Score) health. Calculation modalities and the standard values were carried out according to the manual by Morfeld et al. (25). Higher values on the sum scales reflect better subjective physical and mental health. Standard values can be found in the manual. For the German SF-12, these were taken from the standardization of the SF-36.

#### 2.3.3 Fatigue

Fatigue was assessed using the German version (FS) (26) of the Chalder fatigue scale (27). The scale is a self-report instrument and measures the intensity of fatigue during the last 4 weeks according to 11 items. Seven items relate to the physical component of fatigue, and four items relate to mental fatigue. For example, the physical dimension of fatigue is surveyed with the questions “*Do you have problems with tiredness?*,” “*Do you need to rest more?*,” or “*Do you feel sleepy or drowsy?*,” while the items “*Do you have difficulty concentrating?*,” “*Do you make slips of the tongue when speaking?*,” or “*How is your memory?*” are examples of the mental dimension of fatigue. The items are answered in a four-point response format, for items 1 to 10, *0* = less than usual, *1* = no more than, *2* = more than, and *3* = much more than usual, and for item 11, *0* = better than, *1* = no worse than, *2* = worse than, and *3* = much worse than. The expressions on the two subscales (physical fatigue and mental fatigue) and a total scale score are determined. The evaluation is either dimensional using a Likert scale from 0 to 3 or categorical using a bimodal scale of (0, 1: 0; 2, 3: 1). Thus, evaluations can be made regarding the severity as well as possible case identification. In the present study, a dimensional evaluation was used. Higher total values represent more pronounced fatigue symptoms. In a study using the Chalder fatigue scale, mean fatigue scores of 24.4 ± 5.8 (*n* = 361) and 14.2 ± 4.6 (*n* = 1,615) were found for CFS patients and a “*non-clinical community*” sample presenting to a general practitioner, respectively (28).

#### 2.3.4 Blood analyses

Immediately after sampling, 2 ml of venous blood collected in Lithium-Heparin-Serum Monovettes (Sarstedt, Nümbrecht, Germany), on ice and under light protection, was taken to the mobile lab desk for further processing. Blood samples were processed for the measurement of the superoxide anion (${\text{O}}_{2}^{•-}$) production rate as a surrogate for ROS production and the quantification of oxidative stress-induced DNA strand breaks (single cell gel electrophoresis: “tail moment” in the “comet assay”).

##### 2.3.4.1 Superoxide anion (${\text{O}}_{2}^{•-}$) production

Superoxide anion (${\text{O}}_{2}^{•-}$) production was determined based on electron paramagnetic resonance (EPR) using the VitaScreen^®^ device (Noxygen Science Transfer and Diagnostics GmbH, Elzach, Germany). For this purpose, the device was heated to 37°C to mirror *in vivo* conditions, and 15 μl of blood was pipetted into a light-protected PCR reaction tube. The blood solution was mixed with 15 μl of the spin probe 1-hydroxy-3-methoxycarbonyl-2,2,5,5-tetramethylpyrrolidine (CMH, 400 μmol/L) (Elzach, Germany) diluted in Krebs-HEPES buffer containing deferoxamine and the Na salt of diethyldithiocarbamic acid. The CMH-blood mixture was sucked up using a microcapillary, sealed on one side with sealing wax, and subsequently placed in the resonator of the VitaScreen^®^. After 10 min of reaction, the result was recorded as “cellular metabolic activity (CMA) of ROS in total cells” in nmol/s (29, 30).

##### 2.3.4.2 DNA damage

Oxidative DNA damage was quantified via the determination of DNA strand breaks using single-cell gel electrophoresis (an alkaline version of the “comet assay”) of whole blood samples. Briefly, cell lysis for at least 1 h and slide processing were performed as previously described in detail (31, 32) using alkali denaturation and electrophoresis (0.86 V/cm at a pH ≈ 13) to transform alkali-sensitive parts of the DNA into DNA strand breaks. After staining every slide with 50 μl ethidium bromide (Carl Roth, Germany) under a fluorescence microscope (Olympus, Germany), DNA damage was analyzed using image analysis to determine the mean “tail moment” and the mean “tail intensity” of 100 randomly selected nuclei per slide (two slides each per measurement in each individual) (COMET Assay IV, version 4.3., Perceptive Instruments, Haverhill, United Kingdom) (32, 33). Nuclei with a calculated “tail moment” of <0.1 were qualified as “undamaged” (33).

#### 2.3.5 Statistical analysis

Data were analyzed with the statistic package SPSS (version 28, IBM, United States). The mean differences were tested using the *t*-test for dependent samples or the Wilcoxon test, depending on whether the assumption of a normal distribution was fulfilled. The significance was stated at *p* < 0.05.

## 3 Results

**Figure 1:**
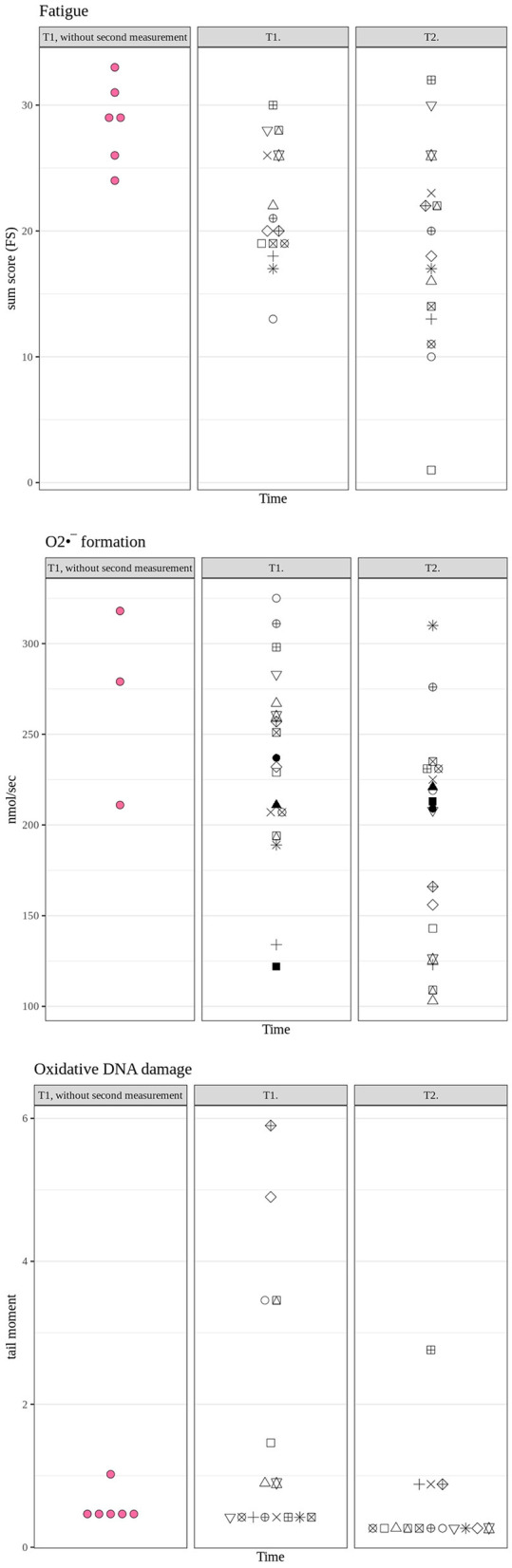
Individual results for the fatigue score **(upper panel)** as well as whole blood ${\text{O}}_{2}^{•-}$ formation rate (in nmol/s) **(middle panel)** and DNA damage (tail moment in the comet assay) **(lower panel)** at T1 and T2. Note that black symbols represent patients for whom *complete* datasets were available at *both* time points T1 and T2, whereas red symbols represent patients for whom data at T2 were not available for all items.

**Figure 2:**
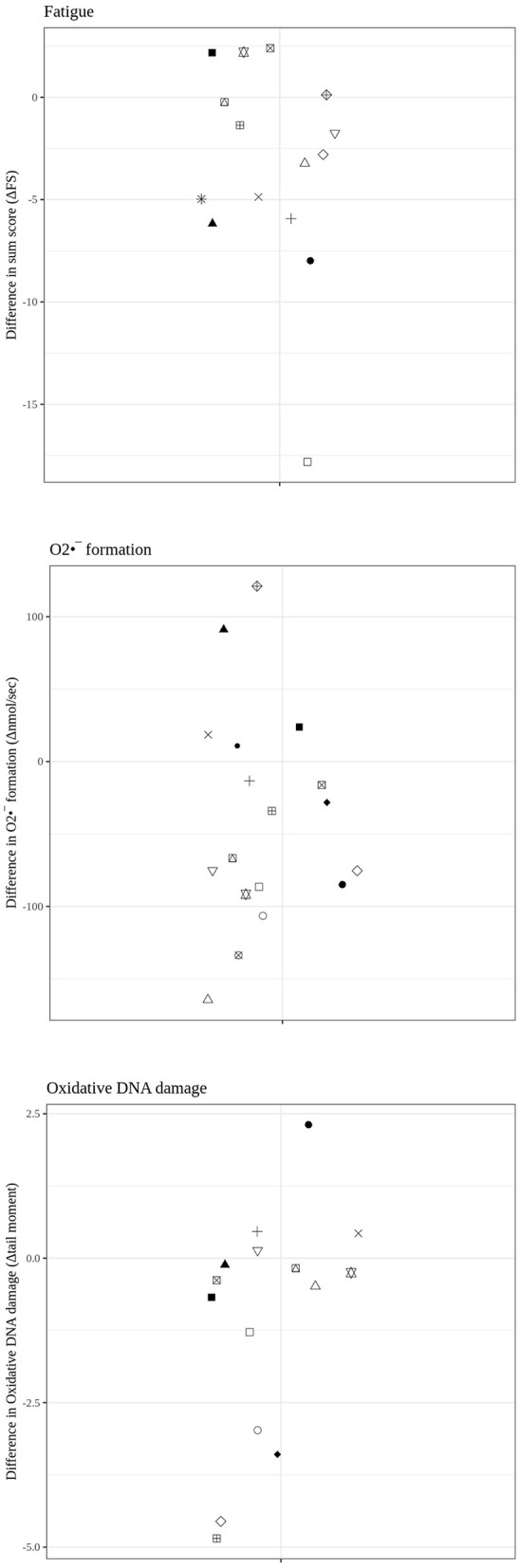
Individual results for the fatigue score **(upper panel)** as well as whole blood ${\text{O}}_{2}^{•-}$ formation rate (in nmol/s) **(middle panel)** and DNA damage (tail moment in the comet assay) **(lower panel)** as difference values between T1 and T2.

Table 1 and Figures 1, 2 summarize the results of the fatigue and mental health parameters as well as ${\text{O}}_{2}^{•}-$ production rate and the quantification of the DNA damage as assessed using the “tail moment” in the “Comet Assay.” While the fatigue severity was significantly reduced from T1 to T2 (Table 1: overall results; Figure 1, **upper panel**: individual findings), the attenuation of the PHQ-15 level just did not reach statistical significance (*p* = 0.054). None of the other psychometric analyses showed any difference. Whole blood ${\text{O}}_{2}^{•}-$ production rate also significantly decreased between the two measurement points (Table 1: overall results; Figure 1, **middle panel**: individual findings), whereas again, the reduction of the “tail moment” just did not reach statistical significance (Table 1: overall results; Figure 1, **lower panel**: individual findings; *p* = 0.053). Figure 2 shows the individual differences between T1 and T2.

**Table 1:** Overall results for fatigue, mental health (SF-12 PCS, SF-12 MCS, PHQ-15, PHQ-9, and PHQ-Stress), whole blood superoxide anion (${\text{O}}_{2}^{•-}$), and DNA damage (“tail moment” in the “comet assay”) at T1 and T2.

	**T1**	**T2**	**Paired *t*-test or Wilcoxon test**	***p*-value**	**Effect size^a, b^**
Fatigue	23.7 ± 5.4 (*n* = 21)	18.3 ± 8.1 (*n* = 15)	*t*_(14)_ = 2.6	0.023	0.42
SF-12 PCS	33.7 ± 9.8 (*n* = 18)	35.5 ± 10.3 (*n* = 15)	*t*_(12)_ = −0.2	0.864	−0.05
SF-12 MCS	37.0 ± 10.3 (*n* = 18)	41.2 ± 13.1 (*n* = 15)	*t*_(12)_ = −0.8	0.435	−0.30
PHQ-15	13.0 ± 5.8 (*n* = 21)	10.1 ± 5.8 (*n* = 15)	*t*_(14)_ = 2.1	0.054	0.31
PHQ-9	9.6 ± 4.5 (*n* = 21)	7.7 ± 4.6 (*n* = 15)	z = −1.2	0.281	0.32
PHQ-Stress	5.6 ± 3.1 (*n* = 21)	4.5 ± 3.1 (*n* = 15)	z = −0.7	0.464	0.19
${\text{O}}_{2}^{•-}$ [nmol/s]	239 ± 55 (*n* = 21)	195 ± 59 (*n* = 18)	*t*_(17)_ = 2.3	0.037	0.70
Tail moment	0.67 (0.36; 1.28) (*n* = 21)	0.32 (0.23; 0.71) (*n* = 15)	z = −1.9	0.053	0.50

Data are presented as mean ± SD or median (interquartile range), respectively, depending on the presence/absence of normal data distribution. Note that the p-values for the paired t-test and the Wilcoxon test refer to the number of measurements available at both T1 and T2. For individual data, see Figure 1. ^a^Cohen's d: Calculation modalities effect size: https://www.psychometrica.de/effect_size.html (t-test), ^b^r = |z/root N|(Wilcoxon test).

## 4 Discussion

The present observational, exploratory, and hypothesis-generating pilot study aimed to assess a possible relationship between oxidative stress and fatigue-like sequelae in hospital employees after a SARS-CoV-2 infection. The main results were that post-COVID-19 fatigue coincides with (i) enhanced ${\text{O}}_{2}^{•-}$ formation and oxidative stress, which are (ii) reduced with attenuation of fatigue symptoms.

The fatigue severity, as assessed using the Chalder fatigue score, was significantly reduced between the two measurement time points. While the fatigue score at T1 (23.7 ± 5.4) was similar to that reported in 361 CFS patients (24.4 ± 5.8) (28), the values at T2 were still higher (18.3 ± 8.1) than in 1,615 control patients (14.2 ± 4.6) in that study. However, in CFS patients, oral oxaloacetate (34), graded exercise (35), and cognitive behavioral therapy (36) had yielded similar reductions of the Chalder fatigue score by approximately five points (35, 36) from 24–26 to 19–21 and 25% (34). Hence, the attenuation of the fatigue score in our post-COVID patients well agrees with reports on various therapeutic interventions in CFS patients.

According to the PHQ-stress score, our patients presented with only a mild stress level at T1. Consequently, given the only minor symptomatic burden, we did not expect a major effect on the PHQ-stress score at T2, and the mean difference was negligible. Both the PHQ-9 score, i.e., the quantification of depressive symptoms, and the PHQ-15 score, i.e., the quantification of somatic symptoms, were only moderate at T1. While the PHQ-9 score did not differ at T2, the PHQ-15 score was attenuated, albeit this effect just did not reach statistical significance (*p* = 0.054). The finding for PHQ-9 well agrees with the assumption that our patients were “mentally healthy,” which is confirmed by the presence of psychotherapeutic treatment in only three patients within the 12 months prior to the investigation. The PHQ-15 score not only addresses mental health but also comprises somatic symptoms that may also be present in CFS patients (37). Hence, given the reduced Chalder fatigue score, it is tempting to speculate that it may have resulted in a reduced PHQ-15 score as well.

In CFS patients, increased plasma peroxide and serum oxidized low-density lipoprotein levels have been reported, suggesting enhanced ROS concentrations [e.g., (38)]. Aggravated oxidative stress resulting from excess ROS production is said to play a role in the development of post-COVID-19 syndrome (39–41). Although, to the best of our knowledge, there is no comparable literature on measuring either ROS formation rate or oxidative stress using the methods shown here, this assumption is in good agreement: the mean ${\text{O}}_{2}^{•-}$ formation rate at T1 was higher than the upper threshold reported for healthy volunteers without an increased ROS production rate [220 nmol/s; (29)] and decreased to levels within the normal range at T2. In addition, the amount of DNA damage as measured using single cell gel electrophoresis and reported as the “tail moment” in the comet assay at T1 (median 0.67) was markedly higher than in various previous investigations of our group in healthy volunteers [median 0.18, 0.23, and 0.30 (31, 32, 42), respectively]. In the present study, at T2, the median tail moment (0.32) had returned to similar values as in these previous studies.

### 4.1 Limitations

The relatively small cohort studied may have precluded more robust, statistically significant results. In addition, due to the observational, exploratory pilot nature of the study, we could not include a control group that did not undergo the educational talk on the typical symptoms of post-COVID-19 syndrome or, in particular, the individualized outpatient procedure. Hence, we cannot discriminate between a possible effect of this procedure and a putative time-dependent resolution of the fatigue symptoms and/or the biological findings. Our study was further limited due to our inability to control possible confounding factors that are well-established to affect DNA damage and/or fatigue (e.g., acute stressors or infections, smoking, nutritional habits, and partial resumption of physical activity). Moreover, to the best of our knowledge, our study is the first to examine fatigue and oxidative cell stress by combining the methods described. Hence, no data are available in the literature that would have supported a case number estimate. Consequently, an *a priori* power analysis was impossible. Finally, our study population was confined to hospital employees, which may cause a selection bias in the recruitment and, consequently, limit the generalizability of the results to a broader population.

### 4.2 Conclusion

Our data suggest a connection between oxidative cell stress and post-COVID-19 fatigue. This possible relationship warrants further investigation so that knowledge can be gained about pathophysiological processes (oxidative stress) in the development of fatigue. This implies psychosomatic treatment options, e.g., mindfulness-based interventions, that stimulate antioxidative targets through psychological and biomolecular mechanisms.

## Data availability statement

The raw data supporting the conclusions of this article will be made available by the authors, without undue reservation.

## Ethics statement

The studies involving humans were approved by Ethikkommission der Bayerischen Landesärztekammer. The studies were conducted in accordance with the local legislation and institutional requirements. The participants provided their written informed consent to participate in this study.

## Author contributions

HH: Data curation, Formal analysis, Funding acquisition, Investigation, Methodology, Software, Validation, Visualization, Writing—original draft, Writing—review & editing. AÖ: Data curation, Formal analysis, Investigation, Methodology, Validation, Writing—original draft. JB: Formal analysis, Writing—review & editing. MG: Data curation, Methodology, Validation, Writing—review & editing. MM: Data curation, Formal analysis, Methodology, Software, Validation, Visualization, Writing—review & editing. FZ: Data curation, Methodology, Validation, Writing—review & editing. BS: Data curation, Formal analysis, Funding acquisition, Methodology, Validation, Writing—review & editing. PR: Conceptualization, Funding acquisition, Project administration, Resources, Supervision, Writing—review & editing. CW: Conceptualization, Funding acquisition, Investigation, Project administration, Resources, Supervision, Writing—review & editing.
